# Supplementary material for: Reward salience but not spatial attention dominates the value representation in the orbitofrontal cortex
Source: Nat Commun. 2022 Oct 22;13:6306. doi: 10.1038/s41467-022-34084-0 (PMC9588087; doi:10.1038/s41467-022-34084-0)
Supplement: Supplementary file 1 — Supplementary Information [file 41467_2022_34084_MOESM1_ESM.docx]

Reward salience but not spatial attention dominates the value representation in the orbitofrontal cortex

**
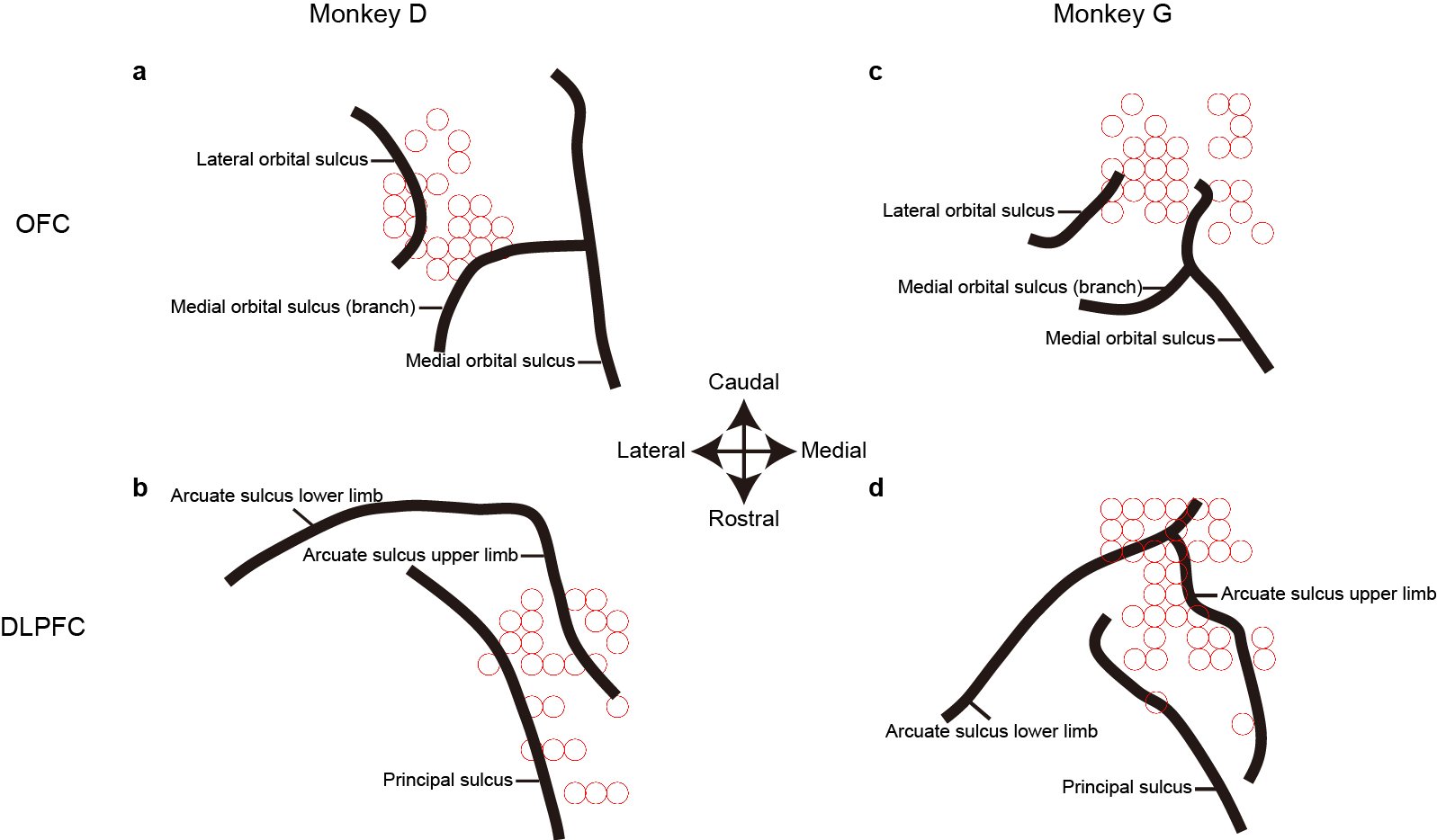
**

**Supplementary Fig. 1.** The schematic diagram of the recording sites. **a.** The recording sites in the OFC in monkey D. **b.** The recording sites in the DLPFC in monkey D. **c.** Same as **a**, but for monkey G. **d.** Same as **b**, but for monkey G. The circles indicate penetration locations. The actual recording sites can be on different sides of a sulcus to what is illustrated here, as the penetrations can be angled.


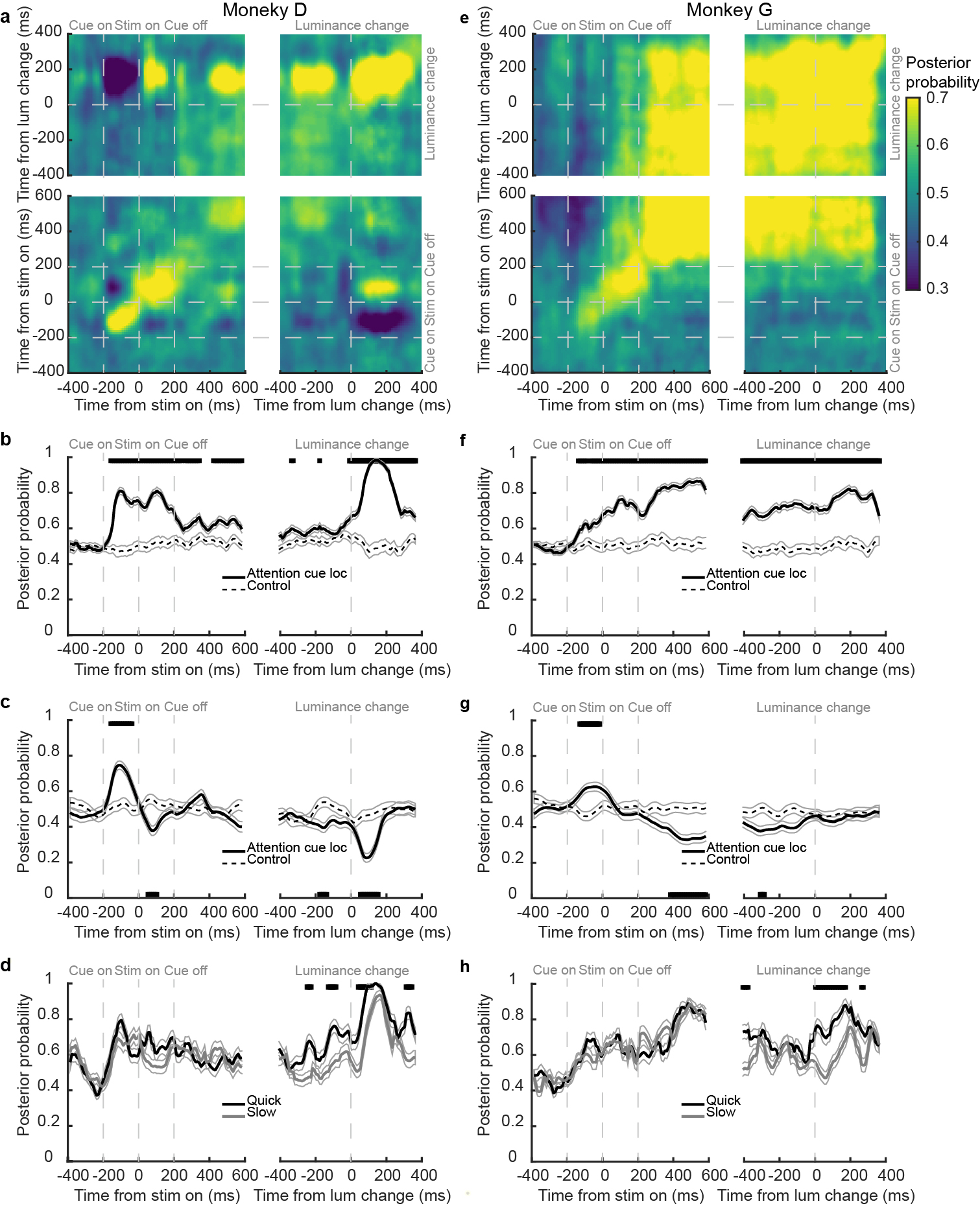


**Supplementary Fig. 2.** Same as **Figure 2**, but for individual monkeys. **a.** The posterior probability of the attention location decoded from DLPFC pseudo-population ensemble activities. **b.** The posterior probability of the attention location from the decoder that was trained and tested with responses at the same time point. Significance was assessed with two-tailed paired *t*-tests (actual data versus shuffled data, at p<0.01 with FDR corrections for multiple comparisons). Thin grey lines represent SEM across trials. **c.** Same as **b** except that the decoder was trained with the mean activities at 50 to 200 ms before the stimulus onset. Significance was assessed with two-tailed paired *t*-tests (actual data versus shuffled data, at p<0.01 with FDR corrections for multiple comparisons). Thin grey lines represent SEM across trials. **d.** The posterior probability of the attention location decoded from the fast (black line) and the slow trials (grey line). Significance was assessed with two-tailed paired *t*-tests (fast versus slow, at p<0.01 with FDR corrections for multiple comparisons). Thin grey lines represent SEM across trials. **a, b, c, d:** monkey D; **e, f, g, h:** monkey G.


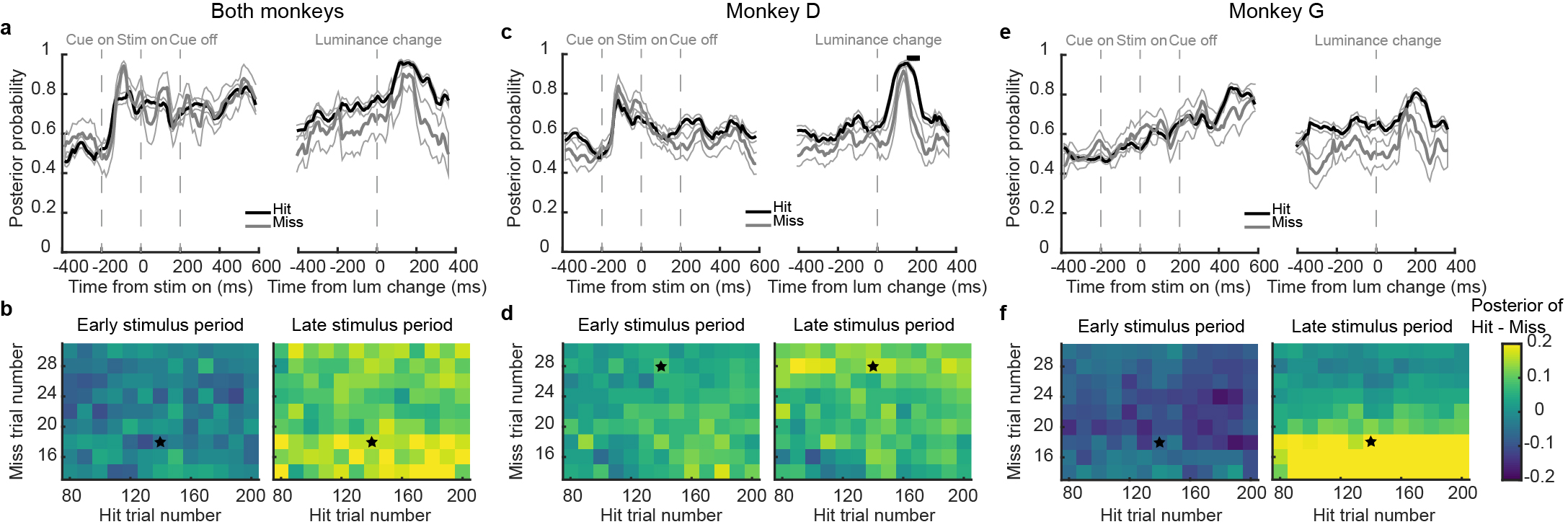


**Supplementary Fig. 3. a.** The posterior probability of the location of the spatial attention decoded from trials with hit responses (black line) and miss responses (grey line) in the DLPFC pseudo neuronal ensemble. Since each session only contained a small number of miss trials, we only used the hit trials to train the decoder and tested it with the un-trained hit trials and the miss trials. **b.** Performances of the decoders depend on the numbers of the hit and the miss trials included for each neuron in the pseudo population ensembles. The posterior probability of the spatial attention location during the early stimulus period (200 to 600 ms after the stimulus onset) and late stimulus period (0 to 400 ms before the luminance change) were shown in the left and the right panels, respectively. Stars indicate the numbers of the hit and the miss trials used in **a**, **c**, and **e**. **a, b:** monkey combined; **c, d:** monkey D; **e, f:** monkey G. Thin grey lines represent SEM across trials.

**
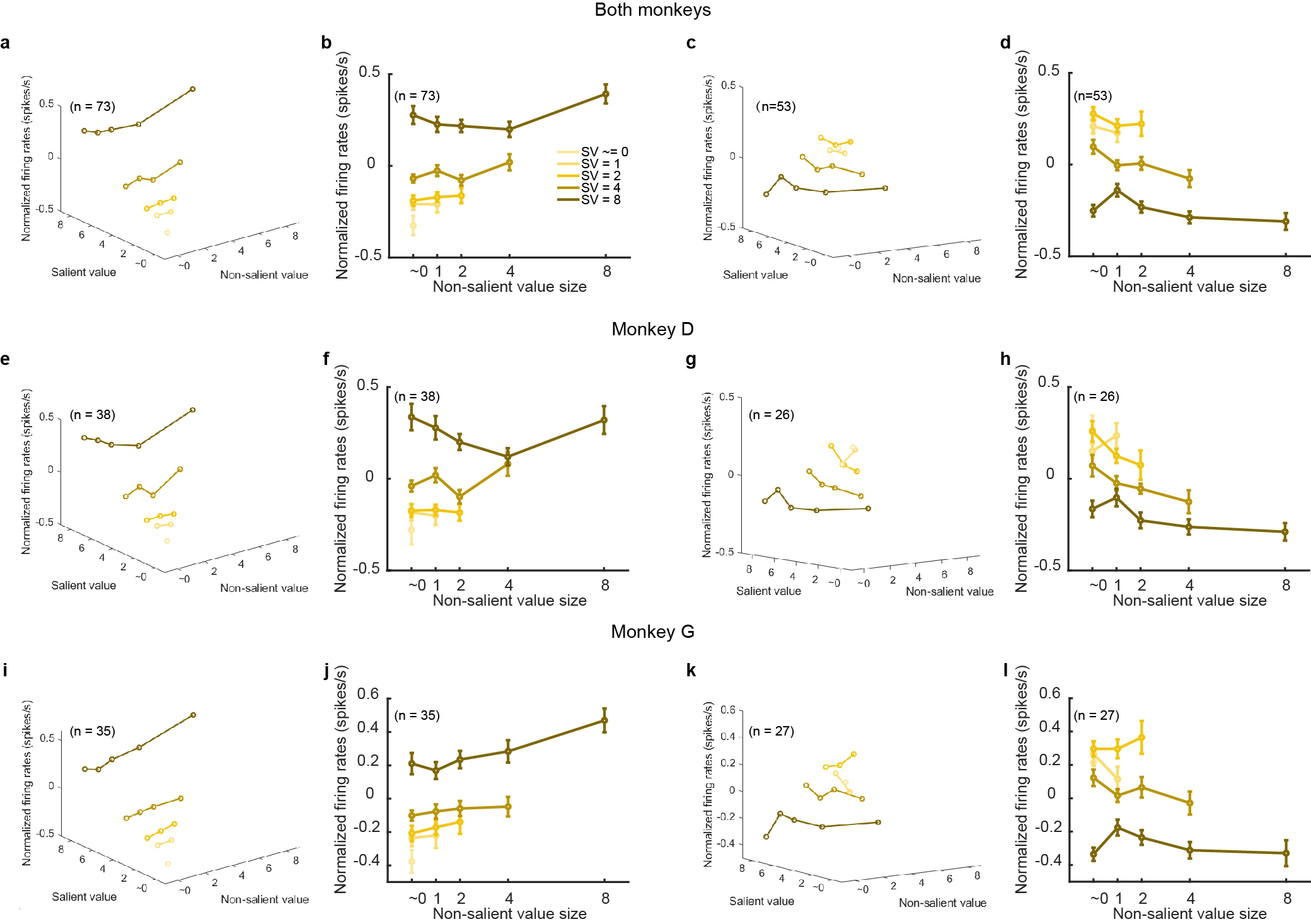
**

**Supplementary Fig. 4.** OFC neurons’ responses were plotted against salient value (SV) and non-salient value (NSV). **a.** Responses of the positively tuned OFC neurons (n=73) plotted against SV and NSV. Colors indicate different SVs. **b.** Responses of the positively tuned OFC neurons were plotted against NSV. Trials are grouped by SV, indicated with colors. **c.** Similar to **a**, but for the negatively tuned OFC neurons (n=53). **d.** Similar to **b**, but for the negatively tuned OFC neurons. **a, b, c, d:** monkey combined; **e, f, g, h:** monkey D (positive-tuned neurons: n=38; negative-tuned neurons: n=26); **i, j, k, l:** monkey G (positive-tuned neurons: n=35; negative-tuned neurons: n=27). The error bars indicate SEM across neurons.

**
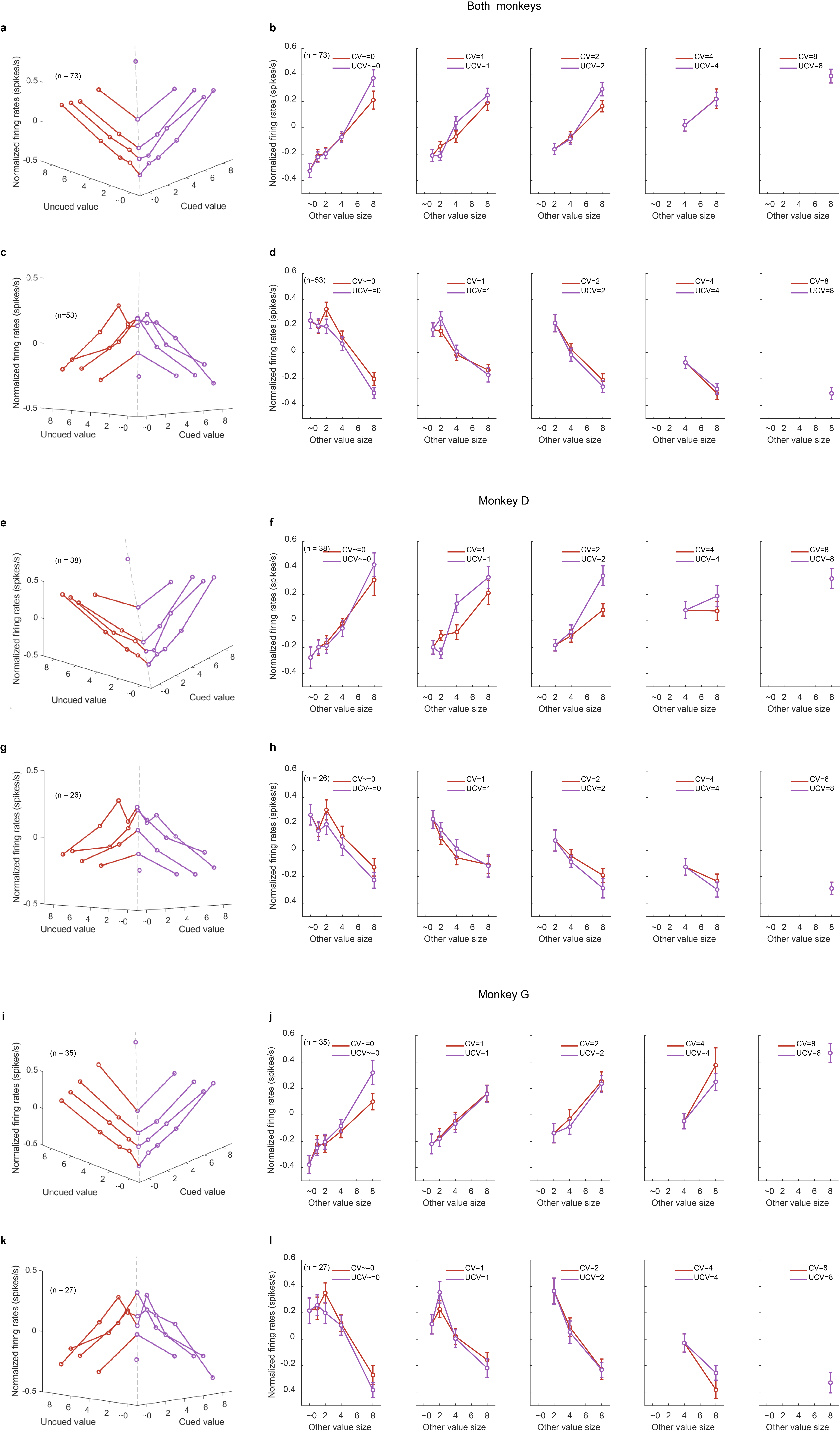
**

**Supplementary Fig. 5.** OFC neurons’ responses were plotted against cued value (CV) and un-cued value (UCV). **a.** Responses of the positively tuned OFC neurons (n=73). Red lines indicate trials with CV less than or equal to UCV, and purple lines indicate trials with CV greater than or equal to UCV. **b.** Responses of the positively tuned OFC neurons under different UCVs (purple) or CVs (red) plotted against the value of the other stimulus. **c.** Similar to **a**, but for the negatively tuned OFC neurons (n=53). **d.** Similar to **b**, but for the negatively tuned OFC neurons. **a, b, c, d:** monkey combined; **e, f, g, h:** monkey D (positive-tuned neurons: n=38; negative-tuned neurons: n=26); **i, j, k, l:** monkey G (positive-tuned neurons: n=35; negative-tuned neurons: n=27). The error bars indicate SEM across neurons.


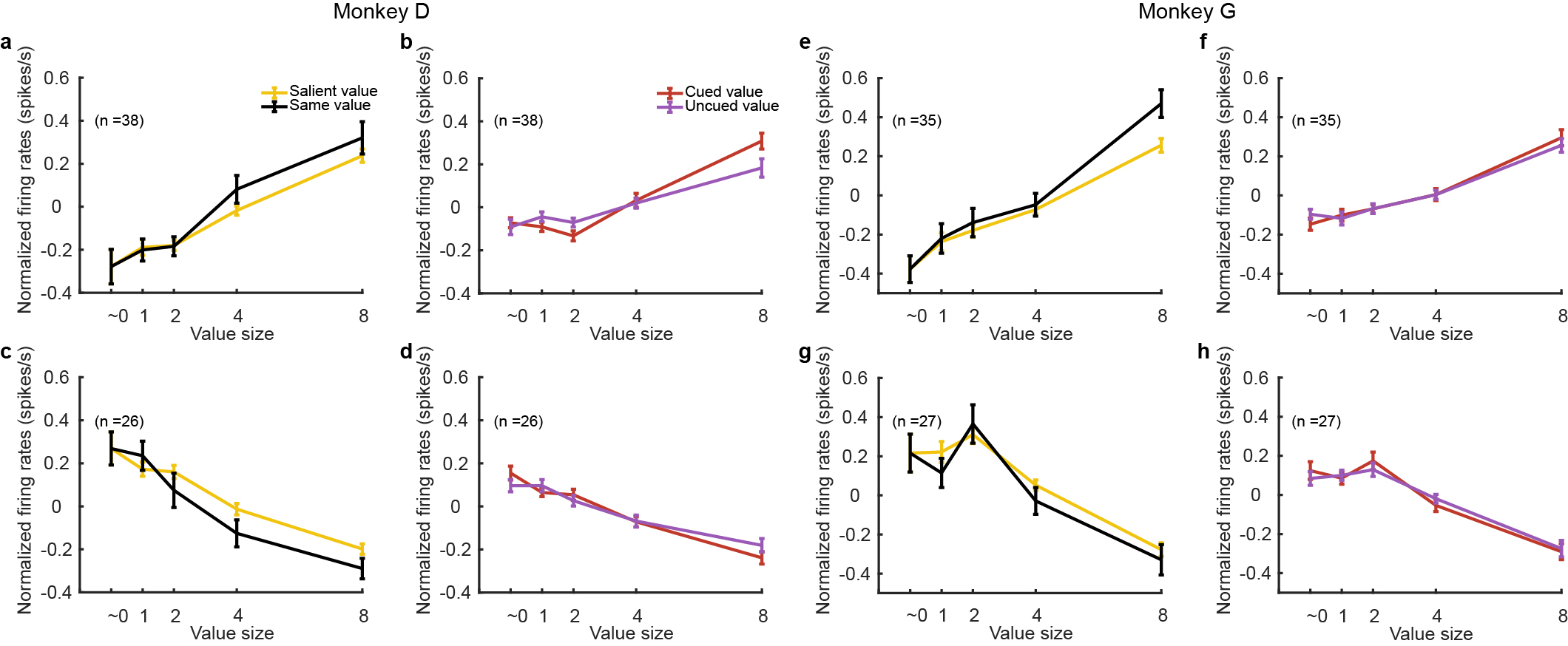


**Supplementary Fig. 6.** Same as **Figure 3**, but for individual monkeys. **a.** The average responses of the positively tuned OFC neurons (n=38) in monkey D. The black trace is based on the trials with the same-reward stimulus pairs. The yellow trace is based on the trials grouped by SV. Two-way ANOVA (group: F_1,374_=0.90, p=0.34; value: F_4,374_=33.4, p<<0.001). **b.** The red trace is based on the trials grouped by CV. The purple trace is based on the trials grouped by UCV. Two-way ANOVA (group: F_1,374_=0.26, p=0.61; value: F_4,374_=47.51, p<<0.001). **c.** Same as **a**, but for the negatively tuned OFC neurons (n=26). Two-way ANOVA (group: F_1,254_=1.59, p=0.21; value: F_4,254_=27.39, p<<0.001). **d.** Same as **b**, but for negatively tuned OFC neurons (n=26). Two-way ANOVA (group: F_1,254_=0.01, p=0.93; value: F_4,254_=48.31, p<<0.001). **e.** Same as **a**, but for monkey G (n=35). Two-way ANOVA (group: F_1,344_=2.57, p=0.11; value: F_4,344_=46.33, p<<0.001). **f.** Same as **b**, but for monkey G (n=35). Two-way ANOVA (group: F_1,344_=0, p=0.96; value: F_4,344_=58.98, p<<0.001). **g.** Same as **c**, but for monkey G (n=27). Two-way ANOVA (group: F_1,264_=0.67, p=0.41; value: F_4,264_=23.97, p<<0.001). **h.** Same as **d**, but for monkey G (n=27). Two-way ANOVA (group: F_1,264_=0.03, p=0.85; value: F_4,264_=47.62, p<<0.001). The error bars indicate SEM across neurons.


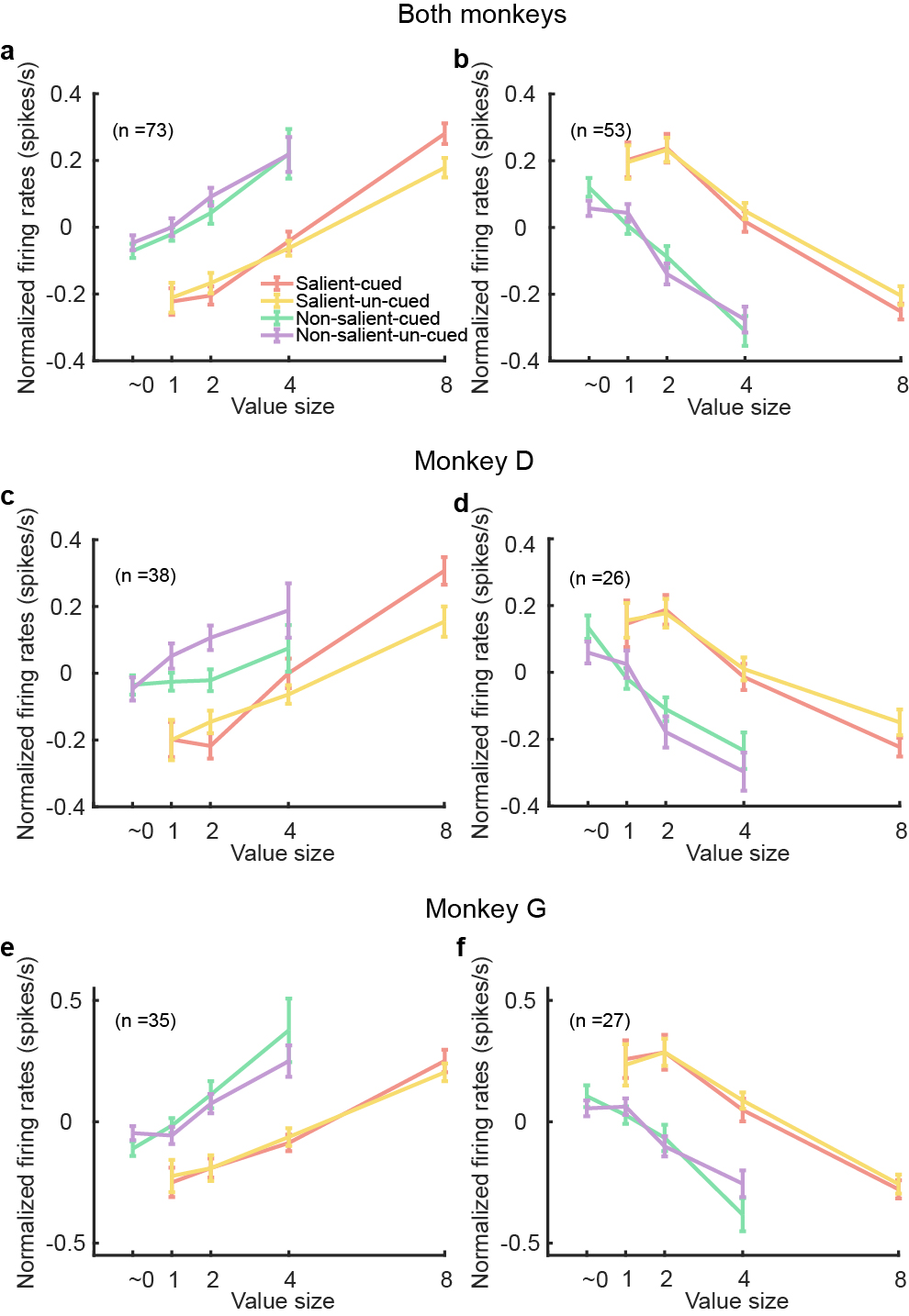


**Supplementary Fig. 7.** Interaction between the cue and the reward saliency’s modulation of the OFC responses. **a.** The average responses of the positively tuned OFC neurons (n=73) during the period between the stimulus onset and the luminance change in both monkeys. Red: trials grouped by SV and CV, yellow: trials grouped by SV and UCV, green: trials grouped by NSV and CV, purple: trials grouped by NSV and UCV. Two-way ANOVA of SV-CV group versus SV-UCV group (group: F_1,579_=0.66, p=0.42; value: F_3,579_=78.63, p<<0.001). Two-way ANOVA of NSV-CV group versus NSV-UCV group (group: F_1,579_=0.70, p=0.40; value: F_3,579_=19.59, p<<0.001). **b.** Same as **a**, but for negatively tuned OFC neurons (n=53). Two-way ANOVA of SV-CV group versus SV-UCV group (group: F_1,419_=0.40, p=0.53; value: F_3,419_=63.71, p<<0.001). Two-way ANOVA of NSV-CV group versus NSV-UCV group (group: F_1,419_=0.21, p=0.65; value: F_3,419_=55.93, p<<0.001). **c.** Same as **a**, but for monkey D (n=38). Two-way ANOVA of SV-CV group versus SV-UCV group (group: F_1,299_=1.34, p=0.25; value: F_3,299_=40.24, p<<0.001). Two-way ANOVA of NSV-CV group versus NSV-UCV group (group: F_1,299_=5.18, p=0.02; value: F_3,299_=4.59, p=0.004). **d.** Same as **b**, but for monkey D (n=26). Two-way ANOVA of SV-CV group versus SV-UCV group (group: F_1,203_=0.59, p=0.44; value: F_3,203_=28.21, p<<0.001). Two-way ANOVA of NSV-CV group versus NSV-UCV group (group: F_1,203_=1.86, p=0.17; value: F_3,203_=28.27, p<<0.001). **e.** Same as **a**, but for monkey G (n=35). Two-way ANOVA of SV-CV group versus SV-UCV group (group: F_1,275_=0, p=0.99; value: F_3,275_=38.16, p<<0.001). Two-way ANOVA of NSV-CV group versus NSV-UCV group (group: F_1,275_=0.66, p=0.42; value: F_3,275_=16.60, p<<0.001). **f.** Same as **b**, but for monkey G (n=27). Two-way ANOVA of SV-CV group versus SV-UCV group (group: F_1,211_=0.05, p=0.82; value: F_3,211_=37.10, p<<0.001). Two-way ANOVA of NSV-CV group versus NSV-UCV group (group: F_1,211_=0.33, p=0.56; value: F_3,211_=29.17, p<<0.001). The error bars indicate SEM across neurons.


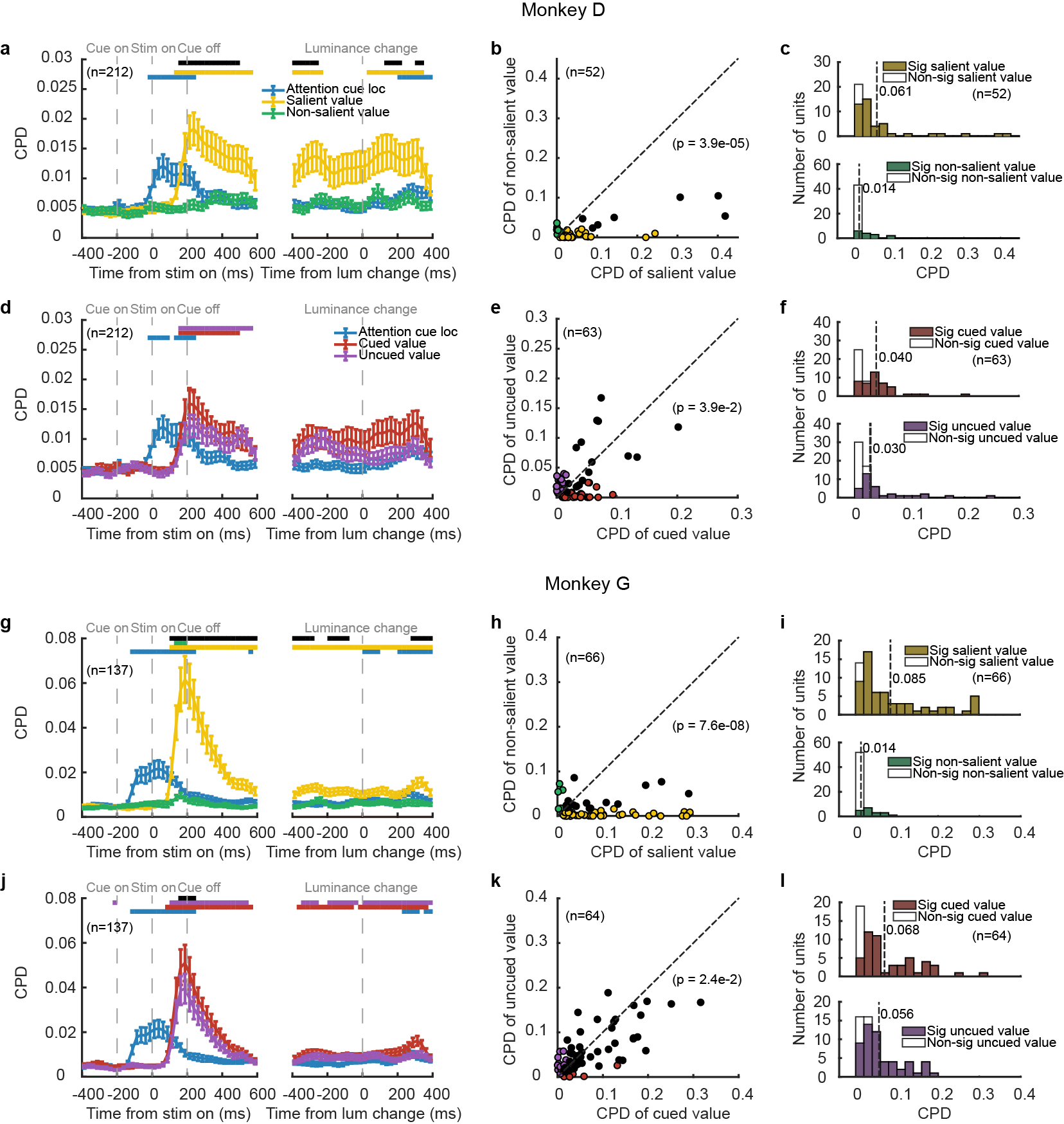


**Supplementary Fig. 8.** Same as **Figure 4**, but for individual monkeys. **a.** OFC neuron firing rates were regressed against attention cue location, SV, and NSV. Plotted is the time course of the population average coefficients of partial determination (CPD). Conventions as in **Figure 4a**. **b.** The CPDs of SV against the CPDs of NSV for individual value-selective OFC neurons. Conventions as in **Figure 4b**. **c.** Top: the distribution of the CPDs for SV of the value-selective OFC neurons. Bottom: the distribution of the OFC neurons’ CPDs for NSV. Vertical dashed lines indicate the mean. Filled bars indicate significant neurons. **d.** Same as **a**, but for CV and UCV. **e.** Same as **b**, but for CV and UCV. **f.** Top: the distribution of the CPDs for CV of the value-selective OFC neurons. Bottom: the distribution of the OFC neurons’ CPDs for UCV. Vertical dashed lines indicate the means. Filled bars indicate significant neurons. **a, b, c, d, e, f:** monkey D; **g, h, i, j, k, l:** monkey G.

**
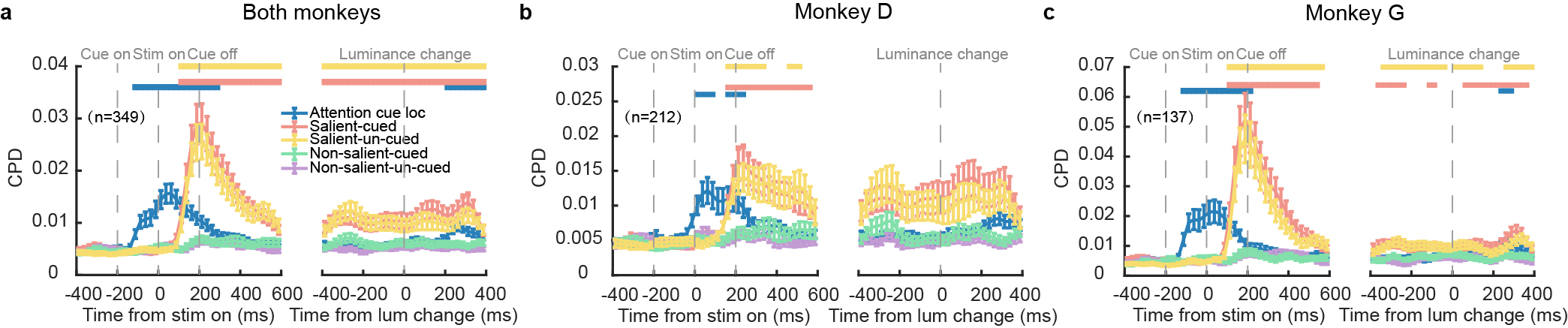
**

**Supplementary Fig. 9.** OFC neuronal responses dominantly encoded salient value. **a.** Time course of average coefficients of partial determination (CPD) for the attention cue location, SV-CV, SV-NCV, NSV-CV, and NSV-NCV in the two monkeys. Significance was assessed with two-tailed paired *t*-tests (p < 0.005, with FDR corrections for multiple comparisons) compared to a baseline computed with the average CPD between 0 and 200 ms before the cue onset and across different regressors. The blue, red, yellow, green, and purple bars at the top indicate the significant CPDs of the cue location, SV-CV, SV-UCV, NSV-CV, and NSV-UCV, respectively. The error bars indicate SEM across neurons (n=349). **b.** Similar to **a**, but for monkey D (n=212). **c.** Similar to **a**, but for monkey G (n=137).


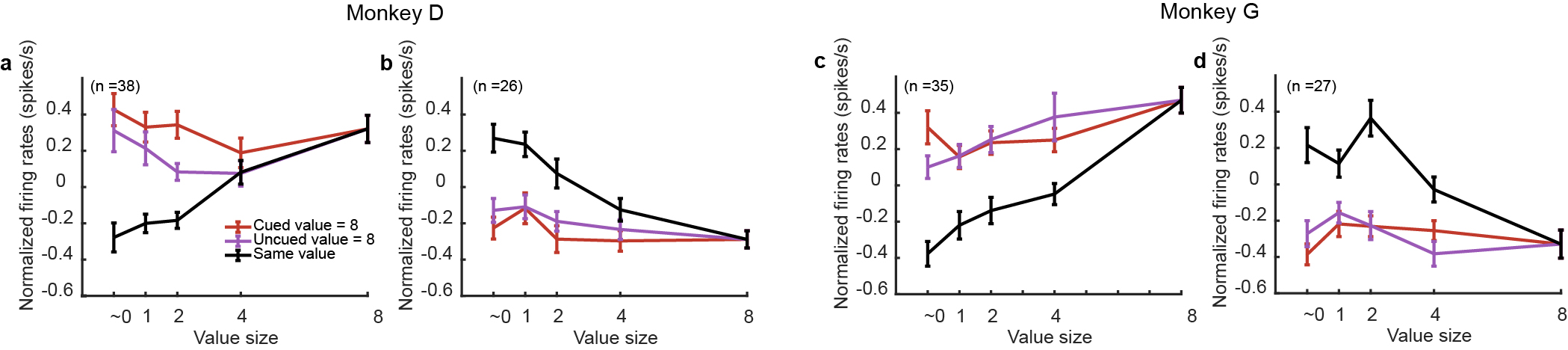


**Supplementary Fig. 10**. Same as **Figure 5**, but for individual monkeys. **a.** The positively tuned OFC neurons’ (n=38) responses in monkey D. A two-way ANOVA (attention location: F_1,374_=5.50, p=0.02; value: F_4,374_=2.57, p=0.04). **b.** The negatively tuned OFC neurons’ (n=26) responses in monkey D. A two-way ANOVA (attention location: F_1,254_=1.86, p=0.17; value: F_4,254_=2.66, p=0.03). **c.** Same as **a**, but for monkey G (n=35). Two-way ANOVA (attention location: F_1,343_=0.08, p=0.78; value: F_4,343_=4.70, p=0.001). **d.** Same as **b**, but for monkey G (n=27). Two-way ANOVA (attention location: F_1,264_=0.06, p=0.80; value: F_4,264_=1.94, p=0.10). The error bars indicate SEM across neurons.


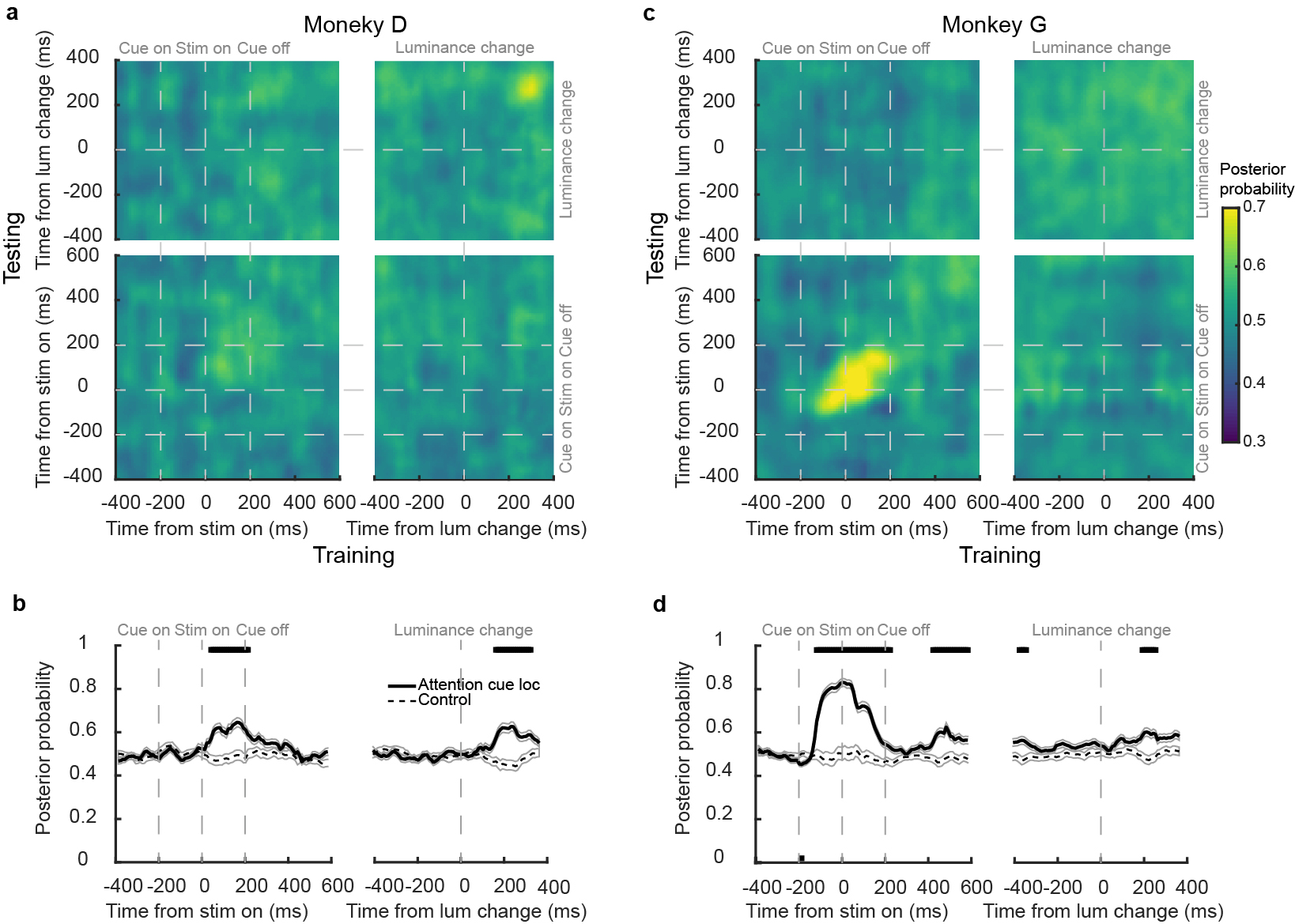


**Supplementary Fig. 11.** OFC did not encode the frame cue location of the monkeys’ attention well after the cue offset. **a.** The posterior probability of the attention location decoded from OFC pseudo-population ensemble activities. **b.** The posterior probability of the attention location from the decoder that was trained and tested with responses at the same time point. Significance was assessed with two-tailed paired *t*-tests (actual data versus shuffled data, at p<0.01 with FDR corrections for multiple comparisons). Thin grey lines represent SEM across trials. **a, b:** monkey D; **c, d:** monkey G.


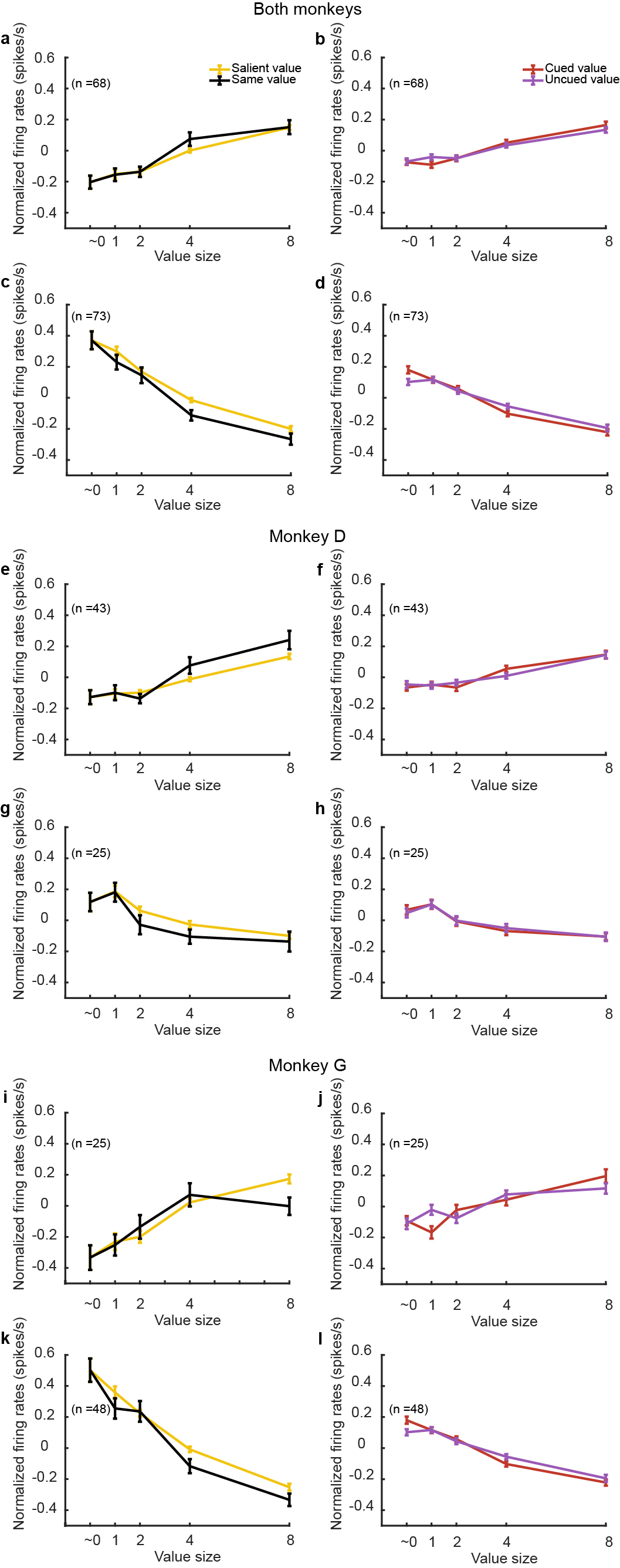


**Supplementary Fig. 12.** Same as **Figure 3**, but for DLPFC neurons. **a.** The average responses of the positively tuned neurons (n=68). The black trace indicates trials grouped by the same-reward stimulus pairs. The yellow trace indicates trials grouped by SV. Two-way ANOVA (group: F_1,674_=0.45, p=0.50; value: F_4,674_=38.05, p<<0.001). **b.** Same as **a,** but for trials grouped by CV (red) and UCV (purple). Two-way ANOVA (group: F_1,674_=0.02, p=0.89; value: F_4,674_=52.96, p<<0.001). **c.** Same as **a**, but for the negatively tuned DLPFC neurons (n=73). Two-way ANOVA (group: F_1,724_=4.24, p=0.04; value: F_4,724_=77.58, p<<0.001). **d.** Same as **b**, but for negatively tuned DLPFC neurons. Two-way ANOVA (group: F_1,724_=0.06, p=0.80; value: F_4,724_=112.17, p<<0.001). **e.** Two-way ANOVA (group: F_1,424_=1.73, p=0.19; value: F_4,424_=24.04, p<<0.001). **f.** Two-way ANOVA (group: F_1,424_=0, p=0.96; value: F_4,424_=34.15, p<<0.001). **g.** Two-way ANOVA (group: F_1,244_=1.92, p=0.17; value: F_4,244_=13.41, p<<0.001). **h.** Two-way ANOVA (group: F_1,244_=0, p=0.98; value: F_4,244_=19.09, p<<0.001). **i.** Two-way ANOVA (group: F_1,244_=0.19, p=0.67; value: F_4,244_=17.54, p<<0.001). **j.** Two-way ANOVA (group: F_1,244_=0.07, p=0.80; value: F_4,244_=20.22; p<<0.001). **k.** Two-way ANOVA (group: F_1,474_=2.97, p=0.09; value: F_4,474_=74.84, p<<0.001). **l.** Two-way ANOVA (group: F_1,474_=0.10, p=0.75; value: F_4,474_=107.29, p<<0.001). **a, b, c, d:** monkey combined; **e, f, g, h:** monkey D (positive-tuned neurons: n=43; negative-tuned neurons: n=25); **i, j, k, l:** monkey G (positive-tuned neurons: n=25; negative-tuned neurons: n=48). The error bars indicate SEM across neurons.


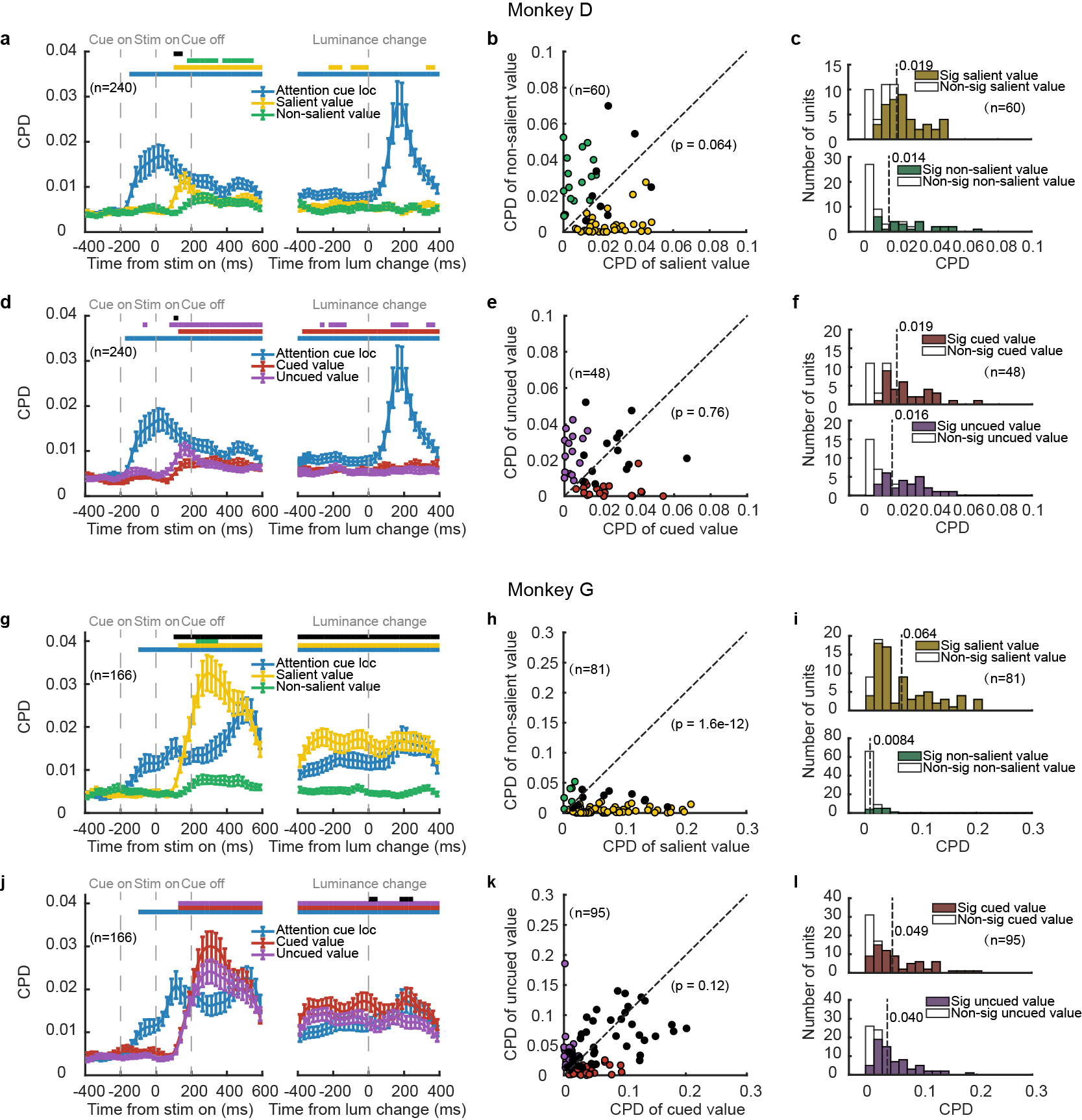


**Supplementary Fig. 13.** Same as **Figure 6**, but for DLPFC neurons. **a.** DLPFC neuron firing rates were regressed against attention cue location, SV, and NSV. Plotted is the time course of the population average coefficients of partial determination (CPD). Conventions as in **Figure 6a**. **b.** The CPDs of SV against the CPDs of NSV for individual value-selective DLPFC neurons. Conventions as in **Figure 6b**. **c.** Top: the distribution of the CPDs for SV of the value-selective DLPFC neurons. Bottom: the distribution of the DLPFC neurons’ CPDs for NSV. Vertical dashed lines indicate the mean. Filled bars indicate significant neurons. **d.** Same as **a**, but for CV and UCV. **e.** Same as **b**, but for CV and UCV. **f.** Top: the distribution of the CPDs for CV of the value-selective DLPFC neurons. Bottom: the distribution of the DLPFC neurons’ CPDs for UCV. Vertical dashed lines indicate the means. Filled bars indicate significant neurons. **a, b, c, d, e, f:** monkey D; **g, h, i, j, k, l:** monkey G.


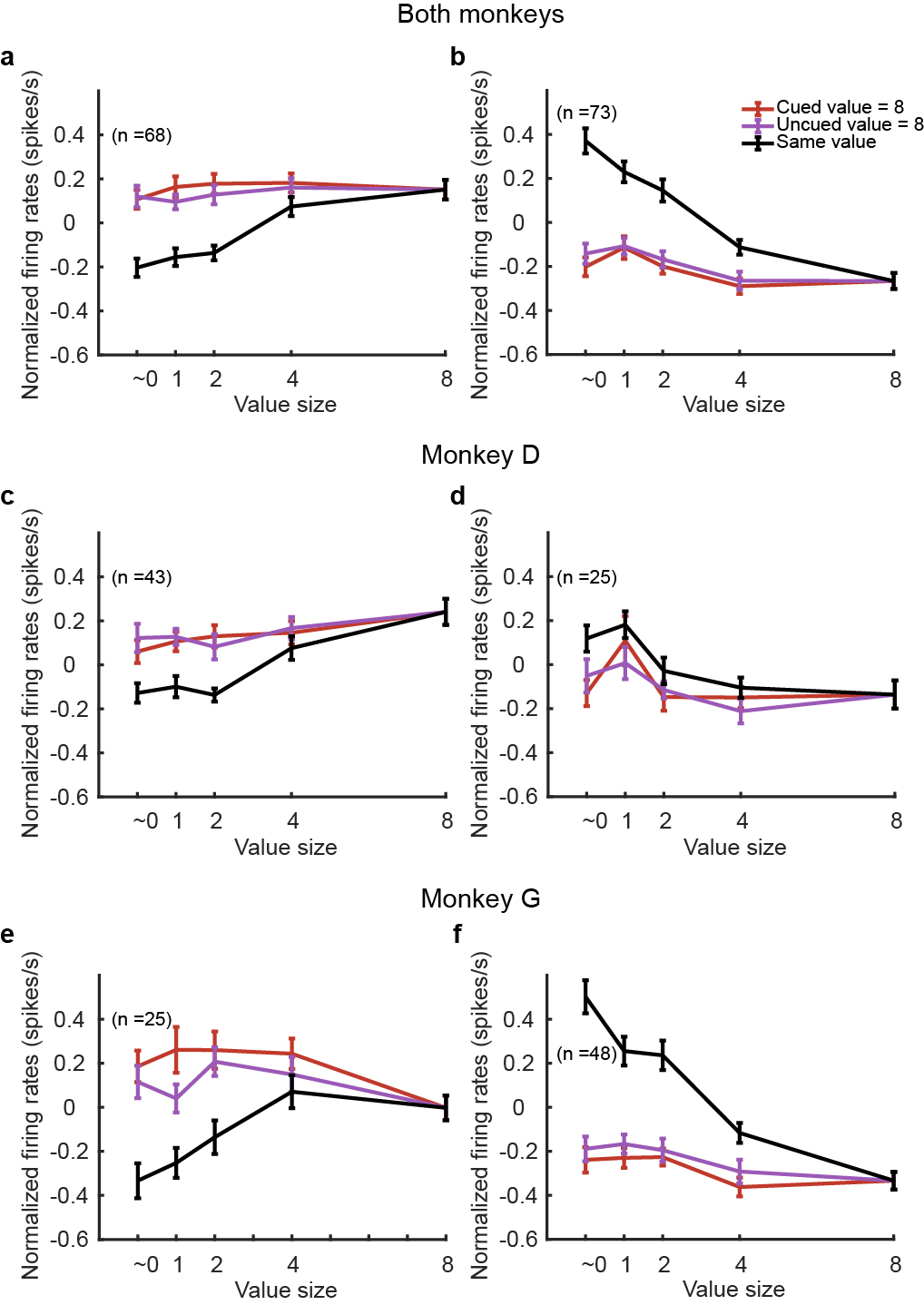


**Supplementary Fig. 14**. Same as **Figure 5**, but for DLPFC neurons. **a.** The positively tuned neurons’ responses (n=68). A two-way ANOVA (attention location: F_1,674_=0.83, p=0.36; value: F_4,674_=0.53, p=0.71). **b.** The negatively tuned neurons’ responses (n=73). A two-way ANOVA (attention location: F_1,724_=0.96, p=0.33; value: F_4,724_=6.09, p=1.0e-4). **c.** Same as **a**, but for monkey D (n=43). Two-way ANOVA (attention location: F_1,424_=0.11, p=0.74; value: F_4,424_=2.55, p=0.04). **d.** Same as **b**, but for monkey D (n=25). Two-way ANOVA (attention location: F_1,244_=0.06, p=0.80; value: F_4,244_=3.69, p=6.1e-3). **e.** Same as **a**, but for monkey G (n=25). Two-way ANOVA (attention location: F_1,244_=3.56, p=0.06; value: F_4,244_=2.99, p=0.02). **f.** Same as **b**, but for monkey G (n=48). Two-way ANOVA (attention location: F_1,474_=2.06, p=0.15; value: F_4,474_=4.06, p=3.0e-3).

**
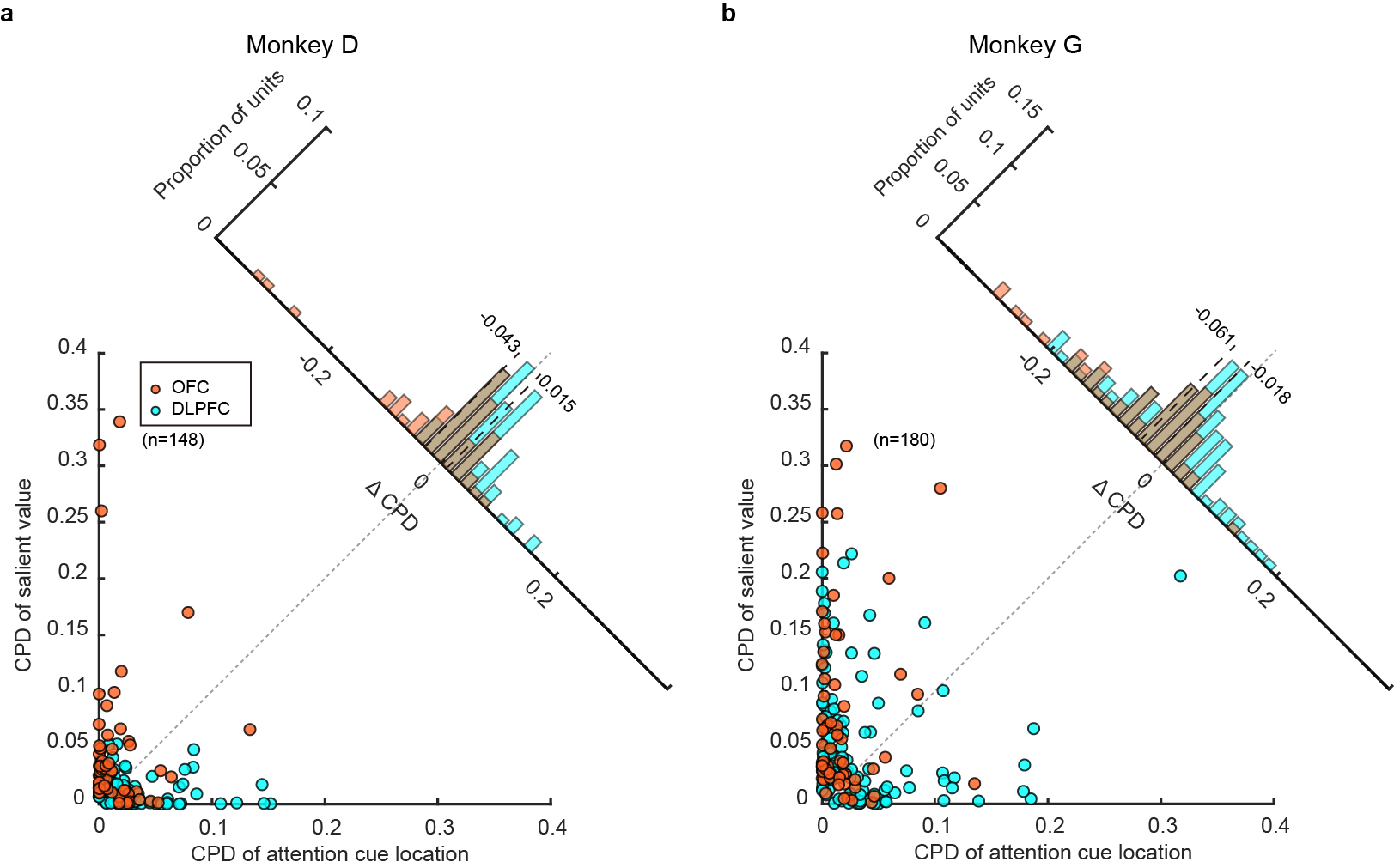
**

**Supplementary Fig. 15.** Comparison of the spatial attention and value signals between OFC and DLPFC neurons in individual monkeys. **a.** The CPDs of the attention cue location are plotted against the CPDs of SV in monkey D. **b**. Same as **a**, but for monkey G. Only the neurons with non-zero coefficients for the attention cue location or SV were plotted (monkey D: n=148; monkey G: n=180. p<0.05, one-sample *t*-test without multiple comparisons).

**
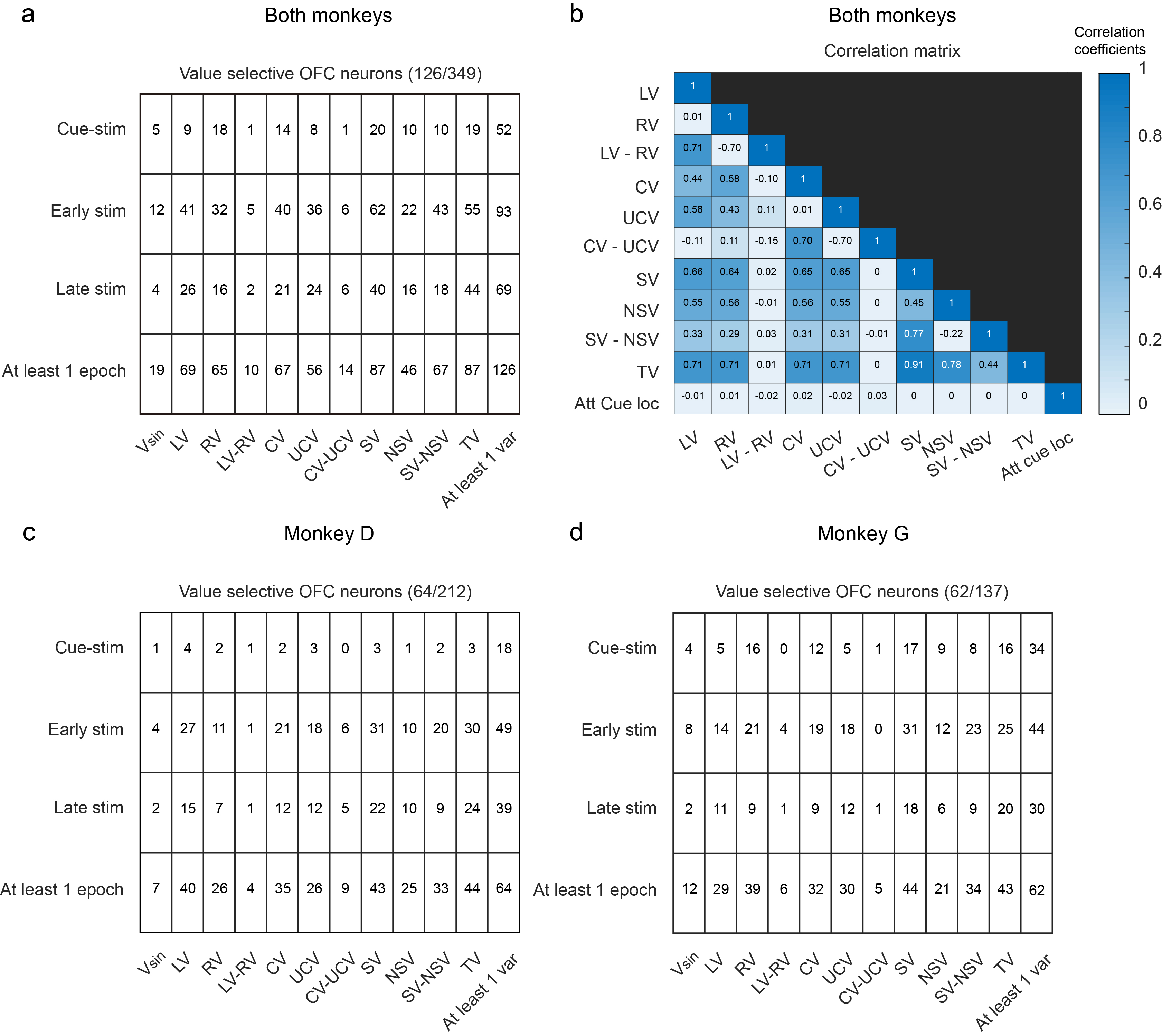
**

**Supplementary Table 1. a.** Number of OFC neurons significantly selective to each variable at each time epoch in both monkeys. The task-related variables include stimulus value in single stimulus trials (V_sin_), left stimulus value (LV), right stimulus value (RV), difference between LV and RV (LV-RV), cued value (CV), un-cued value (UCV), difference between CV and UCV (CV-UCV), salient value (SV), non-salient value (NSV), difference between SV and NSV (SV-NSV), total value (TV) and attention cue’s location (att cue loc) in double stimuli trials. Time epochs include the cue-stimulus epoch (0.0 to 0.2 s after the stimulus onset), the early stimulus epoch (0.2 to 0.6 s after the stimulus onset), and the late stimulus epoch (0.0 to 0.4 s before the luminance change). **b.** Pearson correlation coefficients between each pair of variables. **c.** Same as **a**, but for monkey D. **d.** Same as **a**, but for monkey G.
