# Supplementary material for: Reward salience but not spatial attention dominates the value representation in the orbitofrontal cortex
Source: Nat Commun. 2022 Oct 22;13:6306. doi: 10.1038/s41467-022-34084-0 (PMC9588087; doi:10.1038/s41467-022-34084-0)
Supplement: Supplementary file 3 — Reporting Summary [file 41467_2022_34084_MOESM3_ESM.pdf]

Corresponding author(s): Tianming Yang
Last updated by author(s): Sep 16, 2022

Reporting Summary

Nature Portfolio wishes to improve the reproducibility of the work that we publish. This form provides structure for consistency and transparency in reporting. For further information on Nature Portfolio policies, see our Editorial Policies and the Editorial Policy Checklist.

Statistics

For all statistical analyses, confirm that the following items are present in the figure legend, table legend, main text, or Methods section.

n/a	Confirmed	
☐	☒	The exact sample size (*n*) for each experimental group/condition, given as a discrete number and unit of measurement
☐	☒	A statement on whether measurements were taken from distinct samples or whether the same sample was measured repeatedly
☐	☒	The statistical test(s) used AND whether they are one- or two-sided<br>*Only common tests should be described solely by name; describe more complex techniques in the Methods section.*
☐	☒	A description of all covariates tested
☐	☒	A description of any assumptions or corrections, such as tests of normality and adjustment for multiple comparisons
☐	☒	A full description of the statistical parameters including central tendency (e.g. means) or other basic estimates (e.g. regression coefficient) AND variation (e.g. standard deviation) or associated estimates of uncertainty (e.g. confidence intervals)
☐	☒	For null hypothesis testing, the test statistic (e.g. *F*, *t*, *r*) with confidence intervals, effect sizes, degrees of freedom and *P* value noted<br>*Give P values as exact values whenever suitable.*
☒	☐	For Bayesian analysis, information on the choice of priors and Markov chain Monte Carlo settings
☒	☐	For hierarchical and complex designs, identification of the appropriate level for tests and full reporting of outcomes
☐	☒	Estimates of effect sizes (e.g. Cohen's *d*, Pearson's *r*), indicating how they were calculated

Our web collection on statistics for biologists contains articles on many of the points above.

Software and code

Policy information about availability of computer code

Data collection	Behavioral training was run with MATLAB-based (R2010a) software MonkeyLogic (version 1.0.26). Eye-tracking data was collected using EyeLink 1000. Electrophysiological data were collected using AlphaOmega SnR system (version 2.0.4.5). Spikes were sorted with Plexon Offline Sorter (version 4.5.0).
Data analysis	Behavioral and electrophysiological data were analyzed using MATLAB (version R2019a). The MATLAB built-in functions that were used in this study are noted in Method. The MATLAB custom codes are available at https://github.com/tmyang-lab/reward_salience_in_OFC.

For manuscripts utilizing custom algorithms or software that are central to the research but not yet described in published literature, software must be made available to editors and reviewers. We strongly encourage code deposition in a community repository (e.g. GitHub). See the Nature Portfolio guidelines for submitting code & software for further information.

Data

Policy information about availability of data

All manuscripts must include a data availability statement. This statement should provide the following information, where applicable:

- Accession codes, unique identifiers, or web links for publicly available datasets
- A description of any restrictions on data availability
- For clinical datasets or third party data, please ensure that the statement adheres to our policy

The data used in this study are available at http://doi.org/10.5281/zenodo.7090240.

Human research participants

Policy information about studies involving human research participants and Sex and Gender in Research.

Reporting on sex and gender	N/A
Population characteristics	N/A
Recruitment	N/A
Ethics oversight	N/A

Note that full information on the approval of the study protocol must also be provided in the manuscript.

Field-specific reporting

Please select the one below that is the best fit for your research. If you are not sure, read the appropriate sections before making your selection.

☒ Life sciences ☐ Behavioural & social sciences ☐ Ecological, evolutionary & environmental sciences

For a reference copy of the document with all sections, see nature.com/documents/nr-reporting-summary-flat.pdf

Life sciences study design

All studies must disclose on these points even when the disclosure is negative.

Sample size	406 DLPFC neurons and 357 OFC neurons were collected from two male rhesus monkeys in 171 recording sessions overall. Each recording session contained on average 1381 trials, of which 885 were correct. There was no predetermined number of recording trials, and we kept recording from the same cell until either the monkeys quit working, or we lost the cell. On average, the recording data for each cell contained 547 trials, of which 330 were correct. The sample size in our study is comparable to those in previously published papers (Lin et al., PNAS 2020).
Data exclusions	The offline sorting process excluded neurons with poor isolation from noise or with a lower than 1 Hz firing rate. Data analysis required each neuron to contain at least a certain number of correct trials (see Method). No additional selection criteria for the neurons.
Replication	Behavioral analysis was performed in two monkeys across all the recording sessions. No monkeys failed to learn the behavioral tasks. Single-unit analysis was replicated in all the recording neurons across two brain areas. Pseudo neuronal ensembles were constructed from 200 randomly sampled trials and tested on leave-one-out cross-validations.
Randomization	The animals were chosen based on availability before the start of the experiment. Attention cue location could be either left or right with a predetermined probability (see Method). Visual stimuli for each trial were randomly sampled from the stimulus set. The reward associated with which stimuli were be delivered after the response was randomly chosen from the two presented stimuli. The neurons which were well isolated from noise were recorded. We did not select neurons based on their task selectivity.
Blinding	The experimenters were blind to how neurons selectively tuned to task conditions during the recording and the offline sorting process.

Reporting for specific materials, systems and methods

We require information from authors about some types of materials, experimental systems and methods used in many studies. Here, indicate whether each material, system or method listed is relevant to your study. If you are not sure if a list item applies to your research, read the appropriate section before selecting a response.

Materials & experimental systems

n/a	Involved in the study	
☒	☐	Antibodies
☒	☐	Eukaryotic cell lines
☒	☐	Palaeontology and archaeology
☐	☒	Animals and other organisms
☒	☐	Clinical data
☒	☐	Dual use research of concern

Methods

n/a	Involved in the study	
☒	☐	ChIP-seq
☒	☐	Flow cytometry
☒	☐	MRI-based neuroimaging

Animals and other research organisms

Policy information about studies involving animals; ARRIVE guidelines recommended for reporting animal research, and Sex and Gender in Research

Laboratory animals	Two male rhesus monkeys (Macaca mulatta) aged 11-12 years were used in this study. One weighed 9.3 kg (subject D), the other (subject G) weighted 6.8 kg at the beginning of the training.
Wild animals	No wild animals were used in this study.
Reporting on sex	Two male rhesus monkeys were used in this study. Our findings should not be different in other sex.
Field-collected samples	No field-collected samples were used in this study.
Ethics oversight	All experimental procedures were approved by the Animal Care Committee of Shanghai Institutes for Biological Sciences, Chinese Academy of Sciences (Shanghai, China).

Note that full information on the approval of the study protocol must also be provided in the manuscript.
